# Indian jujube a potential fruit tree to improve the livelihood

**DOI:** 10.1016/j.sjbs.2023.103769

**Published:** 2023-08-05

**Authors:** Nayan Kumar Sishu, Utpal Das, Chinnadurai Immanuel Selvaraj

**Affiliations:** a Department of Biotechnology, School of Bio Sciences and Technology, Vellore Institute of Technology, Vellore 632 014, Tamil Nadu, India; b VIT School of Agricultural Innovations and Advanced Learning, Vellore Institute of Technology, Vellore 632 014, Tamil Nadu, India

**Keywords:** Indian Jujube, Industrial, Livelihood, Nutraceutical

## Abstract

Indian Jujube, also known as Ber or *Ziziphus Mauritiana* Lam., is a fruit-bearing tree endemic to South Asia, including India, Pakistan, Bangladesh, and Sri Lanka. The tree belongs to the buckthorn family and is known for its fruit, a tiny, round, or oblong-shaped drupe roughly the size of a cherry or a small plum. Indian Jujube has been growing for thousands of years. It is a popular fruit throughout the tropical and subtropical regions of Asia, Africa, and South America. Despite the fruit's delicious flavour and health benefits, it is also known for its therapeutic value. Many studies have suggested that various components of ber trees, such as fruit, seed leaves, roots, and flowers, include bioactive substances that demonstrate the potential for antioxidant activity and have anticancer, antibacterial, and antidiabetic effects. Due to the crop's minimal management requirements, it may slow down climate change and the threat of extreme soil and weather conditions, such as drought resistance, strong winds, erosion, high salt, and floods. The main objectives of the current systematic review are to understand Ber's chemical compositions, health benefits, culinary uses, major nutraceutical features, and its function in fostering livelihoods and climatic tolerance.

## Graphical abstract

**Figure:**
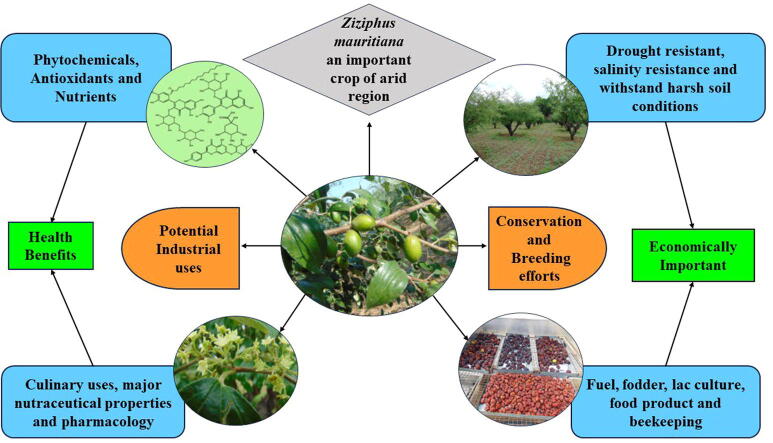

## 1 Introduction

*Ziziphus mauritiana* is one of the traditional ancient fruits-commonly known as Indian jujube or Indian Plum, belonging to the family Rhamnaceae and is indigenous to Southern Asia and Eastern Africa. It is widely cultivated throughout the world. The plant can grow in semi-arid or arid regions and areas with low rainfall, high temperature, high wind, and a wide variety of soils like limestone, laterite, and sandy (Lim, 2012, Singh et al., 2020). It is a hardy tree that can withstand extreme climatic conditions and yield well; thus, its cultivation attracts farmers worldwide. *Ziziphus mauritiana* can grow in soil with neutral or slightly alkaline pH (7.5). However, it can quickly acclimatize in shallow to deep soil like clayey, sandy, and rocky. However, the most favourable soil is sandy loam for good plant growth (Pareek, 2013). It is a spiny shrub or small tree reaching up to 3–15 m with a trunk of about 40 cm or more in diameter.

**Fig. 1:**
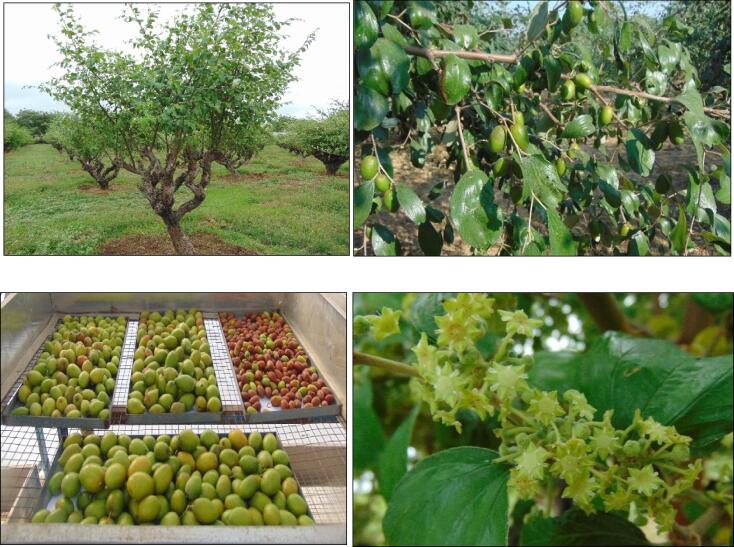
*Ziziphus mauritiana* leaves, flowers, raw and ripen fruits.

The branches have stipular spines, and the ends are bent downwards. The bark is dark grey or dull black. The leaves are simple, ovate or oblong, rounded at both ends, and arranged alternately in two rows. The leaves are adaxially shiny green and abaxially whitish tomentose. The leaves have three basal nerves and two stipular spines; one is long and straight, and the other is short, bent back, and frequently brown (Orwa et al., 2009, Lim, 2012, C.A.B.I UK, 2022). The inflorescence is axillary cyme, 1–2 cm long, with minute greenish-yellow hermaphrodite flowers. The flowers have 3–8 mm long pedicles, five petals, and a calyx with five deltoid lobes. The fruit is an edible drupe, globose or ovoid, initially green, then it becomes yellow, orange, or red on ripening.The fruits of the wild type can grow to a diameter of 2 cm, whereas those of the cultivated type can grow up to or more than 5 cm longer. The fruit has a tuberculate, irregularly furrowed stone-like seed consisting of 6 mm long elliptical red-brown kernels, 1–2 in number. The pictorial representation of the tree, leaves, flower, raw, and ripen fruit are represented (Fig. 1).The fruit is edible and eaten fresh, dried like dates, salted, or pickled. The fruit provides a high source of carotene, vitamins A and C, and fatty acids. Fruits can be macerated in water to create a cool, refreshing drink, and young leaves are cooked as a vegetable in Indonesia (Orwa et al., 2009, Lim, 2012). The wood obtained for Indian jujube is reddish-brown in colour, fine-textured, rugged, and robust. Thus, it is used to construct houses and tools in rural regions. It also makes good firewood (Heuzé et al., 2019).

Indian jujube can be propagated from seeds, *in situ* grafting, and budding using rootstocks. According to reports, the fruit has more minerals, vitamin C, and protein than apples and mangoes while having a higher phosphorus and iron content than oranges (Khera and Singh, 1976, Hussain et al., 2021). It is a rich source of dietary fibre, reducing and non-reducing sugar, calcium, magnesium, potassium, sodium, and phosphorus. The fresh ripened fruit pulp contains carotenoids, fluorides, pectin, citric acid, thiamine, riboflavin, niacin, and ascorbic acid. Maleic acid, oxalic acid, and quercetin are reportedly present in the fleshy part of the fruit (Prakash et al., 2020). Studies revealed that seeds, fruits, bark, leaves, and flowers possess important pharmacological properties. Phytochemical analysis revealed the presence of different bioactives belonging to the category of alkaloids, flavonoids, glycosides, saponins, terpenoids, tannins, fatty acids, and phytosterols (Goyal et al., 2012, Najafi, 2013, Mbahi et al., 2018, Prakash et al., 2020, Butt et al., 2021). An ethnomedicinal study of *Ziziphus mauritiana* revealed that the seed kernels have sedative properties and are used as poultices to stop vomiting and nausea and even relieve abdominal pain during pregnancy. Its formulations are used for wound healing and antidote to aconite poisoning. In the Indian state of Bihar, the seeds are used to treat diarrhea. Leaves of the plant are regarded as an astringent and have diaphoretic properties. Formulation of leaves are used to treat typhoid in children. The bark decoction treats gingivitis, boils, diarrhea, and dysentery. In Ayurveda, the roots of *Z. mauritiana* are used in curing cough, headache, and biliousness. The root powder is applied over the wound and ulcer to get relief. The leaves have anti-obesity and antipyretic properties. Fruits are consumed to improve the digestion and purification of blood (Goyal et al., 2012). Studies suggest that *Z. mauritiana* exhibits antioxidant, anticancer, antidiarrheal, antihyperglycemic, antimicrobial, anti-steroidogenic, wound healing, immunomodulatory, anti-inflammatory and hepatoprotective properties (Dahiru et al., 2010, Goyal et al., 2012, Najafi, 2013, Verma et al., 2018, Prakash et al., 2020, Butt et al., 2021, Ramar et al., 2022). *Ziziphus mauritiana* has an extensive geographic range and can withstand drought and flooding. It is a notable versatile fruit tree of arid and semi-arid areas, is due to its high degree of climatic adaptability, which further plays a crucial role in mitigating soil erosion and desertification (Lal and Dhaka, 2007, Dov et al., 2016). Thus, this review aims to highlight the critical aspects of *Ziziphus mauritiana* in terms of its application in industries, livelihood, and climate resilience.

## 2 Nutraceutical properties

*Ziziphus mauritiana *is a multipurpose plant known to have potential pharmacological activities. Many studies were conducted to understand the different biological properties of varying tree parts, including antioxidant activity, anticancer, antimicrobial, and antidiabetic studies. The plant is a good source of polyphenols, flavonoids, alkaloids, glycosides, tannins, and saponins. Reports indicate that the DPPH and H_2_O_2_ scavenging activity of 250 µg/mL fruit extract of *Ziziphus mauritiana* were 79.5 ± 0.83 % and73.4 ± 0.45 %, respectively (Dureja and Dhiman, 2012). San et al. (2013) reported that the total phenolic and flavonoid content of 50% ethanolic extract of *Ziziphus mauritiana *was found to be 27.62 ± 1.43 mg gallic acid equivalent (GAE)/g extract and 0.74 ± 0.03 mg quercetin equivalent (QE)/g extract respectively. The report suggests that the methanolic extract of Apple Ber (hybrid *Ziziphus mauritiana*) exhibited the highest antioxidant activity, followed by ethyl acetate extract. Higher equivalent dry weight exhibited stronger antioxidant defences against DPPH free radicals (Nigam, 2018). Akanda and Hasan, (2021) reported that the total phenol content was higher in the seed extract. Compared to seed extract, stem extract has higher flavonoids. *Z. mauritiana* stems bark and seed extract showed high antioxidant capacity. Further, the cytotoxicity effect of *Ziziphus mauritiana* seed and stem bark extract in brine shrimp lethality bioassay indicates that the seed extract (LC_50_ = 1.46 μg/mL and LC_90_ = 2.48 μg/mL) was found to be most cytotoxic than stem bark extract.

Reports suggest that using an alpha-amylase inhibition assay, the saponin extract of *Ziziphus mauritiana* leaves demonstrated its significant anti-diabetic properties. The percentage of extract inhibition ranged from 21.2 % to 97.09 % in the alpha-amylase inhibition experiment (Dubey et al. 2019). Perumal et al., (2012) indicate that linoleic acid oxidation was effectively inhibited by *Ziziphus mauritiana *leaf extract, followed by stem bark extract. Further, the cytotoxicity was determined against the Vero cell line, and the IC_50_ value was reported to be 59.78 and 61.47 µg/mL.

As per reports, the methanolic fruit extract of *Ziziphus mauritiana *was found to exhibit the highest cytotoxic effect (70–80%) against HeLa cells (cervical carcinoma cells), which was determined by MTT assay (Beg et al., 2016). As per reports, *Ziziphus mauritiana* fruit extract was found to have a significant antibacterial effect on *Escherichia coli* and *Staphylococcus aureus*, exhibiting a zone of inhibition of 11 mm and 8 mm, respectively. In contrast, fruit extract in chloroform exhibited a zone of inhibition of 10 mm and 12 mm against *E. coli* and *S. aureus*, respectively (Beg et al., 2016). Reports suggest that the *Z. mauritiana* fruit extract-mediated silver nanoparticles at 40 µg/mL dosage exhibited an antibacterial effect against *Bacillus subtilis*, *Shigella boydii*, *Escherichia coli,* and *Salmonella enteritidis* by forming an inhibition zone of 14 mm, 10 mm, 9 mm and 8 mm respectively. Further, the antifungal activity of *Z. mauritiana* fruit extract silver nanoparticles was examined against *Aspergillus niger* and *Trichoderma* species at different concentrations. Results revealed that significant antifungal activity was observed against both strains at a dose of 60 μg/mL of Ag/AgCl-NPs by inducing 100% growth inhibition in the culture plates (Kabir et al., 2020). A study on supplementing *Ziziphus mauritiana* leaf powder with Nile Tilapia (*Oreochromis niloticus*) prevented *Aeromonas hydrophila* infection. It increased the expression of the genes for lysozyme and superoxide dismutase. It enhanced the activity of serum lysozyme and liver antioxidant enzymes. Moreover, the fish's survival rate was significantly higher than the control. The fishes fed with 10 g/kg of the dietary supplement containing *Ziziphus mauritiana* leaf showed improved histopathological parameters that were altered due to *Aeromonas hydrophila* infection concerning the liver, spleen, and kidney, and muscle of fish (El Asely et al., 2020). Various studies have reported the presence of essential bioactives in *Ziziphus mauritiana* (Table 1) and different pharmacological aspects of *Ziziphus mauritiana* (Table 2). These studies make *Ziziphus mauritiana* a promising subject for researchers to study the chemical constituents and extracts of its different parts to understand its use in drug development and mechanism of action concerning various diseases.

**Table 1:** Active Constituents of Different Part of the Ber Plant and their Bioactivities.

**Plant part**	**Active constituents**	**Compound class**	**Structure**	**Activity**
Fruit	Magnoflorine(Soraya et al., 2022)	Alkaloid		Anti-diabetic, anti-inflammatory,neuropsychopharmacology, immunomodulatory, hypotensive, & antifungal activities (Xu et al., 2020).
	Nortropanoline(Soraya et al., 2022)	Alkaloid		Anti-diabetic, anticancer, & antiviral activity(Underlin and Jensen, 2019).
	Zizyberenalic acid(Karim et al., 2019)	Pentacyclic triterpenoid		Cytotoxic activity(Lee et al., 2003).
	5-Hydroxymethylfurfural(Kushwaha et a., 2019)	Furan derivative		Antioxidant, anticancer activity & antiproliferative activity (Zhao et al., 2013).
	D-Allose(Kushwaha et a., 2019)	Aldohexose sugar		Immunosuppressant on allogenic orthotopic liver transplantation(Hirooka et al., 2006),Anti-cancerous(Mitani et al., 2009),Suppresses ROS production in the mitochondria(Ishihara et al., 2011),Neuroprotective effects(Liu et al., 2014).
	Naringenin(Memon et al., 2012)	Flavonoid		Antioxidant, antitumor, antibacterial, anti-inflammatory(Salehi et al., 2019)
	Quercetin 3′-O-glucoside(Memon et al., 2013)	Flavonoid		Antidiabetic and antioxidative effects(Panda & Kar, 2007)
	Rutin(Yahia et al., 2020)	Flavonoid glycoside		Antioxidant, and cardio protective activities(Ganeshpurkar and Saluja, 2017)
	Quinic acid(Soraya et al., 2022)	Phenol		Antioxidant and analgesic effects(Benali et al., 2022)
	Hexadecanoic acid(Soraya et al., 2022)	Long-chain fatty acid		Antioxidant, antimicrobial, anti-inflammatory hypocholesterolemia, Nematicide,&Antiandrogenic (Sheela, &Uthayakumari, 2013)
Seed	Ceanothic acid(Guo et al., 2014)	Triterpenoid		Cytotoxic effect(Lee et al., 1998)
	Betulinic acid(Guo et al., 2014)	Pentacyclictriterpenoid		Anti-HIV, anti-bacterial, anti-malarial, and anticancer activities (Moghaddam et al., 2012)
	Rutin(Yahia et al., 2020)	Flavonoid glycoside		suppress phosphodiesterase, which may be essential for smooth muscle relaxations. (Beretz et al., 1978).
Leaves	Ziziphin(Shivakumar& Mohan, 2012)	Triterpenoid saponin		Taste modifier and anti-sweet activity (Kurihara, 1992)
	Hyperoside(Yahia et al., 2020)	Flavonol glycoside		Anticancer, anti-inflammatory, antibacterial, antiviral, & antidepressant effects(Wang et al., 2022)
	Phytol(Ashraf et al., 2015)	Acyclic Diterpene		Anticancer, anti-inflammatory, antibacterial, antiviral, & antidepressant effects(Islam et al., 2018)
	γ-sitosterol(Ashraf et al., 2015)	Steroid		Anti-diabetic & hypolipidemic activity(Balamurugan et al., 2015)
Bark & Stem	Mauritine C(Goyal et al., 2012)	Cyclopeptide Alkaloid		Anticancer or cytotoxic activity (Adam et al., 2022)
Roots	Zizimauritic acid A(Ji et al., 2012)	Nortriterpens		Antibacterial and cytotoxic activity (Ji et al., 2012)
	Mauritine L(Panseeta et al., 2011)	Cyclopeptide Alkaloid		Antimycobacterial &antiplasmodial activities (Panseeta et al., 2011)
	Ceanothic acid(Panseeta et al., 2011)	Triterpenoid		Antiplasmodial and antimycobacterial activities (Panseeta et al., 2011)

**Table 2:** Pharmacological aspects of extracts, isolates and nanoparticle from different parts of *Ziziphus mauritiana.*

**Plant part**	**Extract/ Component**	**Activity**	**Pharmacology**	**Reference**
Seed	Aqueous ethanolic extract	Antihyperglycemic activity	A dose of 800 mg/kg seed extract and 10 mg/kg glyburide significantly reduced blood glucose level, weight loss, and mortality rate. It improved peripheral insulin sensitivity, enhanced glucose/insulin metabolism, or increased islet of Langerhans insulin release. The results showed that the dosages enhanced glucose tolerance, increasing peripheral glucose utilization in diabetic and non-diabetic mice.	Bhatia & Mishra, 2010
Seed	Aqueous ethanolic extract	Anticancer activity	The extract showed the highest cytotoxic effect against HL-60 cells with an IC_50_ value of 40 µg/mL. Moreover, the flow cytometric analysis determined by staining with annexin V-FITC and PI exhibited that the seed extract induced 18.8% and 61.2% apoptosis at five µg/mL and 80 µg/mL, leading to cell death.	Mishra et al., 2011
Leaf	Ethanol & ethyl acetate extract	Hepatoprotective activity	Ethyl acetate extract (400 mg/kg) and ethanol extract (300 mg/kg) reduced the elevated levels of enzyme markers such as AST, ALT, ALP, and total bilirubin. It increased the total protein levels in Wistar rats with paracetamol-induced hepatotoxicity. Further, histological studies showed reduced inflammation and fatty vacuoles in the liver.	Sawarkar et al., 2009
Stembark	Methanol extract	Anti-diarrheal, Analgesic &Hypoglycemic activities	Stem bark extract showed the highest anti-diarrheal (75.68% defecation inhibition, p < 0.001), analgesic (68.63% writhing inhibition, p < 0.001) and hypoglycemic activity (44.27% blood glucose reduction after 3 h, p < 0.001) at 400 mg/kg body weight dose.	Akanda and Hasan, 2021
Bark	Methanol extract	Antiproliferative activity	Thehighest antiproliferative activity of methanol bark extract was observed against lung cancer (GI_50_ = 28 ± 0.90 μg/mL) and breast cancer (GI_50_ = 29.00 ± 0.90 μg/mL) cells, followed by prostate cancer (GI_50_ = 39.87 ± 0.19 μg/mL) and uterus cancer (GI_50_ = 37.10 ± 0.47 μg/mL)cells by inhibiting cell growth.	Tagne et al., 2015
Leaf and bark	Ethanol extract	Antimicrobial activity	Significantantimicrobial activity was observed for leaf and bark ethanolic extract against *S. aureus, Pseudomonas aeruginosa,* & *Candida albicans.*	Mohamadou et al., 2021
Leaf	Aqueous extract	Antioxidant activity	Pre-treatment of rats with 200, 400, or 100 mg/kg body weight of aqueous leaf extract of *Ziziphus mauritiana* decreased ALT, AST, ALP, and TB and increased catalase, glutathione peroxidase, reductase, and superoxide dismutase activity compared to alcohol administered group.	Dahiru and Obidoa, 2008
Fruit	Aqueous extract	Hepatoprotective activity	*Ziziphus mauritiana* fruit aqueous extract was tested for CCl_4_ liver protection. Treatment with fruit extract (250, 500 mg/kg) or silymarin (100 mg/kg). reduced AST, ALT, ALP, total bilirubin, and cholesterol compared to rats administered with CCl_4_ alone at 250 and 500 mg/kg body weight (p < 0.05).	Dahiru et al., 2010
Root	Dichloromethane extract	Immunomodulatory activity	The dichloromethane root extract of *Ziziphus mauritiana* exhibited immunomodulatory activity, which was performed by chemiluminescence assay by measuring the oxidative burst activity. It was found that 25 µg/mL extract exhibited an inhibition percentage of 12.34 with an IC_50_ value of 55.43 ± 7.9.	Afzal et al., 2017
Seed	Heterotrimericlectin fraction	Immunostimulatory and anti-allergic activity	It increased antibody production and was known to be mitogenic for lymphocytes and splenocytes. It also caused macrophages to produce more lysosomal enzymes, which indicates that both the humoral and cellular arms of immunity are stimulated. It completely prevented anaphylactic shock and Arthus reaction in the *in vivo* model.	Butle et al., 2021
Stem bark	Flavonoid fraction from chloroform extract	Immune-stimulatory activity	It stimulates phagocytic index, lysosomal degranulation, and proliferation of splenocytes and lymphocytes.	Talmale et al., 2014
Leaf	Methanol extract	Analgesic activity& Antipyretic effect	At a dose of 500 mg/kg, the extract significantly reduced the perception of pain brought on by thermal, mechanical, and chemical pain models. At the same dose, paw edema reduces at 0, 1, 2, 3, and 4 h after carrageenan injection. Rats with high body temperature after 24 h of administration of subcutaneous yeast injections showed a significant reduction in body temperature after treatment with methanol extract.	Mohankumar et al., 2022
Fruit	Fruitextract-mediated synthesized Ag/AgCl-NPs	Cytotoxicity	The cytotoxicity against mouse EAC cells was investigated using the MTS assay and the clonogenicity assay. The IC_50_ values for the MCF-7 and EAC cells were 29 and 85 μg/mL, respectively. The NPs induced apoptosis in MCF-7 cells by activating the FAS-mediated pathway, generating ROS, inhibiting colony formation, and increasing activation of FAS, caspase-8, and FADD	Kabir et al., 2020
Seed	Ethanolextract	Hypnotic effect	Oral treatment of the extract at 200 mg/kg substantially enhanced sleeping duration in mice intraperitoneally administered with sodium pentobarbital (50 mg/kg body weight).	San et al., 2013
Fruit	Mucilage fraction	Enzyme inhibitory activity	Themucilage fraction showed an inhibitory action against α-glucosidase (87.14%), acetylcholine esterase (86.89%), α-amylase (70.13%) and tyrosinase (47.01%) enzymes.	Sangeethapriya&Siddhuraju, 2014
Stem bark	Methanol extract	Hepatoprotective activity	The methanolic extract at 400 mg/kg body weight significantly reduced the elevated level of hepato-specific enzymes like SGPT, SGOT, and SALP compared to normal control. The rats administered with plant extract exhibited improvement in the liver structural organization compared to the control.	Yadav et al., 2022
Fruit	Aqueous extract	Wound Healing	Topical application of the extract on excision wounds showed a significant increase in the wound healing rate (p < 0.001) by increasing TGF-β1, VEGF, Type I collagen expression, and suppressing inflammatory markers (TNF-α and IL-1β).	Shady et al., 2022

## 3 Potential industrial use

According to Nyanga et al. (2013), *Z. mauritiana* fruit is essential in terms of nutrition, economics, and society in Zimbabwe. Research studies on chemical analysis of the nutritive composition of *Ziziphus mauritiana* seed revealed that the seed powder is rich in proteins and essential amino acids; moreover, the composition (g/100 g dry weight) of seed powder showed 4.21 ± 0.30 g/100 g of moisture, 2.79 + 0.27 g/100 g of ash, 36.10 ± 0.57 g/100 g of proteins, 11.04 ± 0.88 g/100 g of crude fibres, 27.40 ± 0.11 g/100 g of lipids, and 21.26 ± 0.63 g/100 g of carbohydrates. Mineral composition studies revealed that the seed has a high content of minerals which include 154.79 ± 10.50 g/100 g of Na, 589.08 ± 10.69 g/100 g of K, 585.43 ± 41.29 g/100 g of P, 6.23 ± 0.12 g/100 g of mg, 3.52 ± 0.05 g/100 g of Zn, 1.15 ± 0.14 g/100 g of Mn, and1.21 ± 0.15 g/100 g of Fe (Yerima& Adamu, 2011). Reports suggest that the *Ziziphus mauritiana* leaves powder showed different parameters of nutritional composition in percentage (w/w), which include total ash at 8.02%, acid insoluble ash at 2.72%, Water Soluble Ash at 4.11%, Moisture Content at 7.62%, Crude fibres at 13.08%, and Volatile matters with 0.19% (Gupta et al., 2012). The calcium and phosphorus concentrations range in *Ziziphus mauritiana* (Thai Apple Ber) fruits was 20.48 to 23.50 mg/100 g and 24.08 to 25.25 mg/100 g, respectively. Moreover, the ascorbic acid content varies from 25.60 to 27.87 mg/100, and the vitamin A content varies from 14.80 to 16.08 mg/100 g of fruit (Langthasa et al., 2021). Bioadhesive polymers that adhere to biological surfaces are one of many methods of drug delivery for oral diseases. The adhesion between two materials, where the mucosa is one of the biological surfaces, is called mucoadhesive. The availability of drugs through mucosal surfaces is wider and faster. It enhances medication concentration, permeability, delivery, and tissue protection. Mucoadhesion occurs when interfacial forces hold the drug together on the mucus layer of the mucous membranes. Bioavailability is enhanced by localizing the drug using the mucoadhesive delivery method. Polymers interact with the tissue mucosa, extending contact time and the effect. The mucoadhesive delivery method strengthens patient adherence, facilitates drug administration, improves accessibility, and prolongs residence time, which increases drug absorption and efficacy. Several reports indicate that the fruits and seeds of *Ziziphus mauritiana* can be employed in producing mucoadhesive materials suitable for drug delivery (Singh et al., 2013, Hamedi et al., 2016, Ray et al., 2021). According to the report, proximate composition and mineral elements analysis on the fruits of the *Ziziphus mauritiana* plant showed that the fruit contains moisture (5.16 ± 0.29 mg/100 g), ash (6.16 ± 0.29 mg/100 g), sodium (7.67 ± 0.138 mg/100 g), potassium (306.67 ± 11.55 mg/100 g), calcium (0.33 ± 0.003 mg/100 g), magnesium (0.16 ± 0.005 mg/100 g), and phosphorus (1.58 ± 3.34 mg/100 g)in dry weight, respectively (Keta, 2017). The nutritional composition of *Ziziphus mauritiana* fruit per 100 g edible portion is mentioned (Table 3).

**Table 3:** Nutritional composition of *Ziziphus mauritiana* fruitper 100 g edible portion (EP) (Pareek, 2013, Stadlmayr et al., 2013, Prakash et al., 2020).

**Constituents**	**Fruit per 100 g EP**
Calories (kcal)	5.92
Water (g)	85.2–95.4
Protein(g)	2.4–2.5
Fat (g)	2.8–13
Carbohydrate (g)	8.3–17.0
Fibers (g)	0.2–0.6
Ash (g)	0.2–8.5
Total sugar (g)	5.4–10.5
Reducing sugar (g)	1.4–6.2
Non– reducing sugar (g)	3.2–8.0
Vitamin C (mg)	2.8 – 13.6
Ca (mg)	23–25.6
Mg (mg)	2.0–8.0
Fe (mg)	0.8–1.8
P (mg)	7.0–32.0
Na (mg)	0.8–6.0
K (mg)	265
Zn (mg)	0.03
Mn (mg)	1.6
Cu (mg)	0.01

A report suggests that the ethanol–water extract of *Ziziphus mauritiana* fruit obtained from Ultrasound-assisted extraction (UAE) exhibited high antioxidant activity. It can be used as a natural antioxidant in oil and oil-based products as it reduces the oxidation of oil-based products in comparison to synthetic antioxidants such as tertiary-butylhydroquinone, butylated hydroxytoluene and butylated hydroxyanisole used in the study (Delfanian et al., 2016). Vinegar obtained from the fruit pulp of *Z.mauritiana* contains many antioxidants and exhibits high antioxidant activity (Vithlani and Patel, 2010). *Ziziphus mauritiana* ground seed crude fat was saponified, and then its methyl esters were synthesized and analyzed by GC–MS. The GC–MS reading showed the presence of hexadecanoic acid (7.2%), eicosanoic acid (2.1%), docosanoic acid (1.5%), octadecanoic acid (6.9%), 7-octadecenoic (55.2%), 11-eicosenoic acid (1.9%), and 9,12-octadecenoic acid (25.3%). The saturated and unsaturated fatty acid percentage ratio was 5.3%. Moreover, the GC–MS analysis of an unsaponifiable fraction of seed oil showed the presence of squalene (14%), stigmasterol (23.6%), δ-4-Sitosterol-3-one (6.8%), campesterol (5.8%), γ-tocopherol (4.3%). Thus, *Z. mauritiana *is a good oil source rich in monosaturated fatty acids and can be used to produce food products (Memon et al., 2012). Saponins, known to bind cholestrol are also abundant in *Z. mauritiana* seeds and these can lower the cholestrol level. It also possesses the beneficial phytochemical betulinic acid, which could help develop new medicine formulations. This versatile plant has a variety of possible economic uses, including fuel, fodder, and beekeeping (Mishra et al., 2011). Several studies have highlighted the economic importance and various usage of fruits, seeds, and leaves of *Z. mauritiana*, which can be used further at a larger scale in the industrial sector to produce different products (Table 4).

**Table 4:** Potential Industrial use of *Z. mauritiana.*

**Part of the tree/ component of Ber**	**Uses**	**Property**	**Reference**
Fruit (mucilage)	Production of natural gum	The mucilage obtained from the fruit pulp of Indian jujube had pseudo-plastic properties, water holding capacity (11.77 g dry weight), and good oil absorption ability (4.96 g oil/g dry weight); thus, it can be employed in the production of natural gums-related products.	Thanatcha and Pranee, 2011
Fruit pulp	Ethanol production	Conditions like a temperature of 30 °C, pH of 6, and yeast (8.0 g) or fruit pulp (20 g) were ideal for ethanol generation. A concentration of 63 g/L of ethanol was produced in such parameters. *Saccharomyces cerevisiae* (NA33) had to be added to boost the rate and yield of fermentation because the control (without *S. cerevisiae*) vessel revealed a low fermentation rate.	Togarepi et al., 2012
Fruit	Production of fermented beverages	It was found *S. cerevisiae *strains and non-*Saccharomyces *species like *Pichia kudriavzevii*, *Saccharomycopsisfibuligera*, and *Pichia fabianii* that have been isolated from *Ziziphus mauritiana* fruit and their traditionally fermented fruit pulp are suitable starter cultures for production of flavour compounds for fermented products.	Nyanga et al., 2013
Gum isolated from fruit pulp	Mucoadhesive material	The yield % of gum isolated from the fruit pulp was 38.56% at room temperature; it dissolves readily in hot water and partially in cold water. The gum has better mucoadhesive strength than Carbopol 934 and Hydroxypropyl methylcellulose (HPMC) and thus can replace synthetic mucoadhesive polymers and polysaccharides.	Ray et al., 2021
Seed (mucilage)	Mucoadhesive tablet	The mucilage fraction obtained from the seed of *Ziziphus mauritiana* showed substantial mucoadhesion strength. It was found that 3% w/v mucilage has shown mucoadhesion strength comparable to 1 % w/v Carbopol 934P. Thus, it can be employed in forming additives to design oral mucoadhesive tablets.	Gangurde et al., 2012
Fruit extract	Prevention of oxidation of oil-based products	Extract (600 ppm) added to soyabean oil exhibited more thermo-oxidative stability of soybean oil, low carbonyl compounds formation in soyabean oil during the frying process, reduced level of peroxide value in oil, reduced production of conjugated compounds, reduction in the hydrolysis of oil and maximum oxidative stability index of soyabean oil in comparison to synthetic antioxidants.	Delfanian et al., 2016
Dietary fibres from fruit peel	Use in food product	The dietary fibres from the fruit peel by the freeze-drying process exhibited effective DPPH scavenging properties, vitamin C content, total phenolic content, and higher oil-holding capacity. Thus, the dietary fibre fraction of Indian jujube can be employed in various food products.	Sarkar et al., 2022
Leaves	Soap formulation	For the formulation of antioxidant soap, 0.3% leaf extract of *Ziziphus mauritiana* was used as the foaming agent. The best soap formulated showed 1.09 g/mL of density, pH of 9.8, 25.50% of several fatty acids, 1.2462% of free fatty acid, 2.52% of neutral fatty, and foam stability of 83.95%.	Widyaningsih et al., 2022
Fruit pulp	Food product (Biscuit)	The fruit pulp can be used for making biscuits using the steamed sandwich method; the product was better with crude fibre, protein, and carbohydrates. Thus, the fruit pulp biscuit can fulfill nutritional requirements by serving as a useful food product.	Abubakar et al., 2017
Seed	Oil production	Seeds of *Z. mauritiana* L. contain a significant quantity of oil rich in monounsaturated fat, with unique minor components like γ-tocopherol and stigmasterol.	Memon et al., 2012
Fruit	Preparation of flour	Flour with particle size under 125 µm showed more soluble carbohydrate (37.74 ± 0.03 % DM), protein (0.33 ± 0.01 % DM), pectin (2.49 ± 0.01 % DM), and good antioxidant activity (21.95 ± 0.68 µg DPPH/100 g DM). Thus, it could be employed to make a cake with a firm texture, light colour, and good antioxidants and nutrients.	Dairou et al., 2014
*Ziziphus mauritiana* leaves mediated synthesis of copper and nickel nanoparticles	water purification	Copper and nickel nanoparticles adsorbed on filter paper strips are used in biological water purification. It was found effective against pathogenic coliform, thus showing its antimicrobial effect.	Naveed et al., 2019
Adsorbents synthesized from leaves	Bioremediation	Novel bio-sorbents synthesized from branches and leaves of *Capparis decidua* (CDB) and *Ziziphus mauritiana *(ZML) are used for the remediation of potentially toxic cadmium (Cd(II)) ions from wastewater.	Bilal et al., 2021

## 4 Climate resilience

According to reports, the Indian native fruit *Ziziphus mauritiana* is incredibly drought-resistant. It makes up a large portion of the indigenous vegetation in the “Thar desert” of India. It may be successfully grown even in the most vulnerable tropical and subtropical ecosystems. India has planted 90,000 acres of enhanced Ber trees, with an average production of 8.34 tonnes per hectare (Awasthi and More, 2008). Studies revealed that cultivators in the northwest part of India preferred growing *Z. mauritiana* or Ber for its ability to withstand harsh climatic and soil conditions. Even though there are challenges in growing ber regarding access to the markets, poor soil quality, high salinity, and poor access to fresh water, 80% of farmers have been practicing cultivating ber as a crop since the plants require much less attention. However, the yield is affected; thus, it can be managed by adopting better agriculture technology (Singh et al., 2020).

The report suggested that *Z. mauritiana* showed significant fruit yield after being grown under harsh conditions such as arid soil and irrigation done with brackish water containing a salt concentration of 3500 ppm. A study conducted from 2013 to 2018 observed that fruit yield increased over time, rising from its lowest point in the fruiting season of 2013–14 to its highest point in 2017–18. The highest yield/plant recorded was 55.66 kg/plant in 2017–18, followed by 44.95 kg/plant. After harvesting of fruits, pruning was found to be crucial every year (Pathan et al., 2020). It was reported that Ber (*Ziziphus mauritiana *L.) was cultivated in lysimeters artificially salinized with magnesium sulfate, sodium chloride, magnesium chloride, and calcium in salt tolerance. A salinity of 20 dSm^−1^ electrical conductivity was too high for any plants to survive. It was observed that with increasing salinity (15 dSm^−1^) while potassium concentration (425 mg/100 g dry weight) in leaf tissues dropped whereas, the level of sodium (1930 mg/100 g dry weight), calcium (2490 mg/100 g dry weight), Magnesium (1550 mg/100 g dry weight), and Chloride (1360 mg/100 g dry weight) concentrations significantly rose. While lower production and fruit set resulted from higher salinity, fruit quality was unaffected. The investigation's findings indicate that Ber can grow in saline soils with electrical conductivity (EC) up to 11.30 dSm^−1^ (Hooda et al., 1990). According to reports, hexose sugars were significantly higher during drought stress due to changes in sugar metabolism. This suggests that altered solute partitioning may play a significant role in *Ziziphus mauritiana's* ability to withstand drought. Further, it was observed that 0.7 MPa reduced osmotic potential at full turgor according to pressure–volume analyses. In contrast, leaves under drought stress had their osmotic potential reduced by ∼ 1 MPa at turgor loss. Along with osmotic adjustment, bulk tissue elastic modulus (wall rigidity) increased by 65% under progressive dryness, which led to turgor loss in both stressed and unstressed leaves at the same rate (Clifford et al., 1998).

## 5 Conservation and breeding efforts of *Z. mauritiana*

The researcher needs to familiarize themselves with the floral biology of the crop before beginning any breeding program. Depending on the type and agro-climatic conditions, the ber blossoming season in India extends from early June to late November. The length of the flowering period varied from 68 to 94 days overall, depending on the cultivar. The growth of the current season is mainly producing flowers. However, October is when most of the fruit sets. Most kinds do not produce fruit themselves (Pareek et al., 2007). Ber's breeding program's primary goals are to create cultivars with traits such as early maturity, plentiful yield, appealing fruit colour, smaller seed size, firm texture, high soluble content, and excellent eating quality. For biodiversity conservation and future utilization on farm cultivation, germplasm of superior and indigenous *Z. mauritiana* varieties/accessions are essential. The major national and International Institutes which conserves the germplasm and genetic resources of *Z. mauritiana* are Central Arid Zone Research Institute, Jodhpur, Rajasthan, Central institute of Arid Horticulture, Bikaner, Rajasthan, Harayana Agricultural University, Hisar, Harayana and he International Centre for Underutilized Crops (ICUC-IWMI), Colombo, Srilanka.

Various salient features of different ber varieties in India, including biotic and abiotic stress resistance among cultivars, are mentioned (Table 5, Table 6).

**Table 5:** Salient features of different ber varieties in India.

**Sl. No.**	**Variety**	**Salient features**
1	Umran	Average fruit weight is 40–80 g, yield per tree is 200–220 kg, late ripening, and the fruit has excellent storing and transportation qualities.
2	Kadaka	Widely grown in Maharashtra, resistant to drought, Yield: 70–75 kg/tree suited to the export market.
3	Chihuahua	This variety semi-tall, midseason tree with spreading branches. Fruits are ovate-oblong and weigh 16.8 g, dimension 2.9 cm × 2.1 cm.
4	Dandan	Non-spiny, medium to large in size, decent in quality, and easily maintained, late bearing.
5	Sanaur-2	It is a selection from Sanaur village of Patiala district. The pulp's TSS is 15–18%, 45 kg/tree is the average yield. able to withstand powdery mildew.
6	Illaichi	The name 'Illaichi' is derived from the fruit's cardamom-like shape. Each fruit weighs 6 g and measures 2.05 by 1.85 cm. The annual average output is 115 kg per tree.
7	Seb	Each fruit weighs around 30 grammes. The tree grows erect and produces 40–45 kg per tree.
8	Jogia	Hard-seeded and rosette-forming habit of growth.
9	Mehrun	Shining yellow to red - brown fruits produce high-quality, soft-seeded upon dehydrated.
10	Manuki	Medium size and susceptible for powdery mildew
11	Gola	Average weight of 14–17 g, 17–19 percent TSS; 0.46–0.51 percentage acidity, production of 80–100 kg per tree.
12	Sanaur-6	Measures 4.45 × 2.18 cm. The average annual production is 80 kg per tree, with a longer shelf life.
13	Goma Kirti	Early flowering and harvest by three weeks to Umran, good yield per plant (35.6 kg) and storage quality.
14	Thar Sevika	It was created from the hybridization of Seb and Katha. Thar Sevika is a cultivar that matures quickly. Average fruit output is 30–32 Kg/tree.
15	Thar Bhubharaj	A variety of native material from the Bhusavar region of the Bharatpur district in Rajasthan, with an average output potential of 30–36 kg per tree. With a TSS content of 22-23° Brix, the fruits are particularly juicy and delicious.
16	Thar Malti	Late maturity (fruit ripens 145–150 days from fruit set), outstanding output (65–70 kg/plant), and disease resistance. Fruit yield: 65–70 kg/plant.
17	CAZRI Ber 2018	Developed via seedling selection from the var. Gola seedling population, average fruit weight is 19.26 g, pulp stone ratio is 12.33.
18	Kaithli	Average weight180 g, 18% TSS; 0.5% acidity; pulp soft and sweet. Average yield 100–150 kg per plant.
19	Katha phal	Average weight is 10 g, 23% TSS; 0.77% acidity; yield, 80––100 kg per tree. Late in season.
20	Gular Bashi	TSS 18.8% when yellow, 22.4% after turning brown. Stone medium to thin, funnel-shaped, easily, separated from the flesh. Late bearing.
21	Kheera	Medium to large, oval, with a beak; pulp and soft with sweetness. TSS 19.8%. Late, heavy bearer, and of average keeping quality

**Table 6:** Biotic and Abiotic stress resistance among cultivars.

**Traits**	**Cultivar**
Tolerant to fruit fly	Illaichi, Thar Sevika, Thar Bhubhraj, Bawal Selection-1, Bawal selection-2
Resistance to fruit fly	Tikadi, Meharun
Tolerant to Powdery mildew	Bawal Selection-1, Thar sevika, Thar Bhubhraj, Sanaur-5, Kathaphal, safedRohtak, Gola, Seb, Meharun
Resistance to Powdery mildew	Tikadi, Khavaspura
Tolerant to Frost	Mahrawali, ZG-3
Resistance to Frost	Tikadi, Khavaspura
Salt tolerance	*Z. rotundifolia, Banarsi Karaka* > 50% (60.5 ESP, 20.25 dsm^−1^)

### 5.1 Breeding strategy for *Z. mauritiana*

• Offspring trials that replicated across the environments range where *Z. mauritiana* is grown included many accessions.
• Selection of best promising accessions considering qualitative and quantities characters to improve production.
• Ensure control of pollination to improve the cross between the populations.
• After the cross-produced progeny can be replicated and evaluated to ensure that chosen trees ought to make up a sizable share of the breeding population.
• Vegetative propagate of the tree and domesticate the set of selected clones.

## 6 Livelihood generation

According to reports, *Z. mauritiana* thrives in the challenging conditions of the Thar Desert, including dry weather, arid soil, salinity stress, and inadequate water irrigation. Consequently, it will benefit the Thar people in combating climate change, food shortages, and hunger (Pathan et al., 2020). Once the fruit ripens, it is consumed raw in India. The fruit may also be used to produce pickles and can be properly dried out in the sun to extend its shelf life. The leaves are also valuable as fodder because of their nutritional richness and quick regeneration. According to research, silkworms are fed leaves as nourishment. For the lac insect, *Kerria lacca*, which feeds on the leaves' juice and coats them with an orange-red resin, *Z. mauritiana* is grown as a host plant. In baking and jam-making, fruit powder derived from *Z. mauritiana* is utilized. Green, unripe fruits make the sauce, pickles, and jellies (Goyal et al., 2012). As per reports, he primarily uses *Z. mauritiana* in nutrition, forage, and medicines. Its fruits are consumed fresh as such and processed into various products, such as porridge, traditional cake, jam, and alcoholic and non-alcoholic beverages. Under high heat and little rainfall, the tree thrives and produces well. Growing Households in arid and semi-arid areas may benefit from *Z. mauritiana* by having lessened food and financial insecurity, especially in light of recent climate change effects that have led hot, dry zones to spread out. For instance, in Zimbabwe, the fruit is picked in the Zambezi Valley in large numbers and marketed across the country and even beyond. (Maruza et al., 2017). Reports suggest that the lac insect strain, namely *Kerria lacca* (*rangeeni and kusmi*), can be cultivated on *Z. mauritiana*; it was observed that the productivity of lac is high, and there is no adverse effect on the tree if harvesting of crop, pruning, and coppicing is done appropriately. In India, 93 million Ber trees are available, and they can be employed for lac culture. Leaves of *Z. mauritiana *can be used as fodder since it contains high crude protein, crude fibre, starch, and minerals. The tree parts can be used as fuel as the sapwood has 4900 kcals/kg, and timber as the hardwood has a density of 535–1080 kg/m3 (Yogi et al., 2017). As per reports *Z. mauritiana* (Masau) fruits are consumed and sold for a profit in markets to generate an income in Zimbabwe. After exposure to the sun for a few hours, this is used to make a beverage called “mahewu” in Zimbabwe (Nyanga et al., 2008). As per the report of Newaj et al., (2017) there are potential fodder tree-based agroforestry systems, including *Ziziphus mauritiana*, that can boost production, positively affect microclimate, minimize soil degradation, restore soil fertility, and diversify income-generating opportunities.

## 7 Conclusions and future prospects

The whole plant of *Ziziphus* has greater utilization, either edible or inedible, and has been reported in different studies as represented in this review. The active constituents present *in Ziziphus *fruit possess anti-diabetic, anti-inflammatory, and anti-cancerous properties. For year-round availability, the fruits can be processed and dehydrated. They are the source of compounds such as rutin, ceanothic, and betulinic acid, which can treat various illnesses affecting human health. Ziziphin, Hyperoside, Phytol, and γ-sitosterol may be extracted from leaves and used industrially.

Moreover, it provides a significant source of protein on a dry-weight basis, making it a good choice for animal feed. Because of its high economic return, cheap cultivation costs, and greater tolerance to endure drought stress and soil alkalinity, *Ziziphus* is becoming increasingly popular among fruit farmers and may one day serve as a source to stop soil erosion and desertification. Under limited rainfall and high heat conditions, the tree thrives and produces well. Growing *Z. mauritiana* could solve food, nutritional, and economic problems, considering the current climate change effects that have caused the expansion of hot and dry zones. The tree can be used for many things, including making furniture, poles for different purposes, animal feed, medicines, nectar-producing flowers, and much more. The fruit can be processed into various goods, including non-alcoholic and alcoholic beverages, traditional cake, porridge, and jam, and is consumed raw, dry, or fresh. Jujube research should respond more precisely to business, markets, and government needs, and it may even actively develop and lead these demands. Research goals should be more advanced and diverse. Improved rootstock and germplasm evaluation and screening strategies must be adopted to establish high-efficiency propagation systems. Ensuring year-round supply needs to include a variety of cultivars with varying maturation periods, staggering in harvesting, dehydration, and long-distance cold transportation. Thus, it will be essential to focus on developing this crop soon to export it to the non-traditional source of income.

## Declaration of Competing Interest

The authors declare that they have no known competing financial interests or personal relationships that could have appeared to influence the work reported in this paper.
